# Commentary: Leucine-rich Repeat Receptor-Like Gene Screen Reveals that *Nicotiana* RXEG1 Regulates Glycoside Hydrolase 12 MAMP Detection

**DOI:** 10.3389/fgene.2019.00162

**Published:** 2019-03-07

**Authors:** Si Wu, Wei Wang, Xiangru Xu, Xiaolin Wu

**Affiliations:** State Key Laboratory of Wheat and Maize Crop Science, Collaborative Innovation Center of Henan Grain Crops, College of Sciences, Henan Agricultural University, Zhengzhou, China

**Keywords:** genome-wide VIGS screen, leucine-rich repeat (LRR) receptor-like gene (protein), microbe-associated molecular patterns (MAMPs), *Nicotiana benthamiana*, pattern-recognition receptors (PRRs), plant immune response, response to XEG1 (RXEG1) protein, virus-induced gene silencing (VIGS)

Innate immunity against pathogen infection by membrane-localized receptors is evolutionarily conserved among eukaryotes (Palma et al., 2007). In plants, innate immunity is essential for defending against harmful microbes that cause dramatic losses in agriculture. Activation of plant immunity is generally determined by pattern-recognition receptors (PRRs). In contrast to mammals, all known plant PRRs are localized at the cell surface (Macho and Zipfel, 2014). There, they detect potentially harmful microbes by recognizing microbe-associated molecular patterns (MAMPs) (Boller and Felix, 2009; Böhm et al., 2014). The diversity and number of MAMPs recognized by individual plant species is likely larger than previously thought (Brunner and Nürnberger, 2012; Zhang et al., 2013; Böhm et al., 2014; Macho et al., 2014). Thus, characterizing PRRs in plants is key to plant-microbe interaction studies.

PRRs such as receptor-like kinases (RLKs) and receptor-like proteins (RLPs) (Shiu et al., 2004; Fischer et al., 2016) play crucial roles in plant immunity, growth, and development. Plant genomes contain hundreds of such receptor-like genes, most of which encode proteins with extracellular leucine-rich repeat (LRR) domains (Shiu and Bleecker, 2003; Shiu et al., 2004; Diévart et al., 2011; Sakamoto et al., 2012), but only a few PRRs with LRR domains have been identified (Sun et al., 2013; Hind et al., 2016; Tang et al., 2017; Wang et al., 2018). Therefore, an efficient approach for quickly identifying PRRs is important for understanding plant innate immunity and developing disease-resistant plants.

Recently, Wang et al. (2018) developed a high-throughput virus-induced gene silencing (VIGS)-based toolkit for characterizing LRR receptor-like genes on a genomic scale (Figure 1A). The approach was demonstrated on the plant model *Nicotiana benthamiana*, a solanaceous plant and a close relative of tobacco. As a result, 257 tobacco rattle virus–based constructs were generated to silence all 403 identified genes that encode predicted membrane-localized LRR-RLPs and LRR-RLKs in *N. benthamiana*. Moreover, Wang et al. (2018) successfully identified Response to XEG1 (RXEG1), an LRR-RLP that specifically recognizes the glycoside hydrolase 12 protein XEG1. Their study demonstrated that this genome-wide silencing assay can quickly identify new immune receptors to help mine and utilize crop resistance resources. VIGS has also been successfully developed for monocots (Yuan et al., 2011; Liou et al., 2014; Liu et al., 2016). In view of the importance of monocot cereals such as rice, wheat, and maize—and great yield losses due to microbial attacks—it would be worthwhile to evaluate this method for cereal crops.

**Figure 1:**
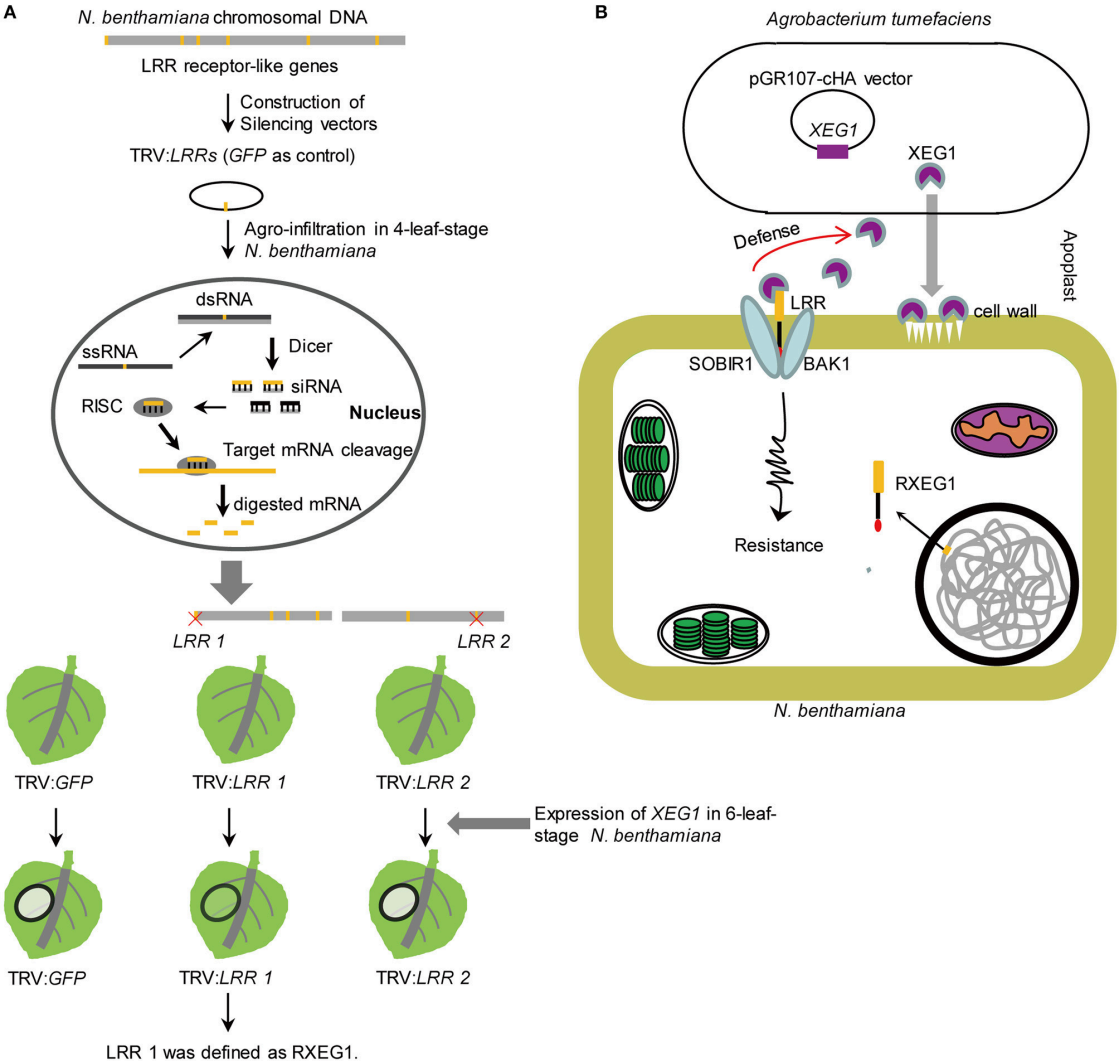
Identification of RXEG1 in *N. benthamiana*. **(A)** VIGS-based approach for identification of PRRs. The 403 LRR receptor-like genes, which encode proteins with both LRR and TM domains, were identified in *N. benthamiana* genome. LRR receptor-like genes were cloned into the modified gene silencing vector pTRV. *Agrobacterium tumefaciens* strain carrying 257 silencing vector*s* inflitrated into 4-leaf-stage *N. benthamiana*. Co-expressing GH12 protein XEG1 that was identified from *Phytophthora sojae* in 6-leaf-stage TRV scilencing *N. benthamiana*. XEG1-included cell death was compromised in leaves treated with RXEG1. **(B)** A possible model of RXEG1's role in defense to XEG1. *Phytophthora* infects plants early by secreting a glycolytic enzyme XEG1 to attack the cell wall. The receptor protein RXEG1, identified by Wang et al. (2018), can recognize XEG1 of Phytophthora infection. Subsequently, RXEG1 was found to be a key factor for cell necrosis and defense response after XEG1 recognition. Activation of RXEG1 could significantly improve plant resistance to *Phytophthora* infection. This figure was made based on Wang et al. (2018) and a news report (http://news.njau.edu.cn/2018/0210/c70a92967/page.htm).

The VIGS-based approach for identification of PRRs has advantages over methods that rely on map-based cloning and Arabidopsis T-DNA insertion lines (Zipfel et al., 2006; Jehle et al., 2013; Zhang et al., 2013; Albert et al., 2015): (1) VIGS can be easily performed on *N. benthamiana*, which are amenable to highly efficient VIGS and protein expression (within one month); and (2) VIGS in *N. benthamiana* avoids gene function redundancy and allows for simultaneous silencing of multiple homologous genes (Wang et al., 2018). Thus, silencing efficiency can be evaluated by analyzing gene expression and alterations in plant growth. The silencing efficiency of 230 LRR receptor-like genes (i.e., 43 LRR-RLP and 187 LRR-RLK genes) is > 60% (Wang et al., 2018).

In addition to identifying and characterizing PRRs, elucidating the mechanisms by which PRRs perceive microbial attack will significantly advance our understanding of plant innate immunity. The comprehensive and intensive work of Wang et al. (2018) revealed how *N. benthamiana* recognizes XEG1, a widely distributed MAMP in microbial taxa (Figure 1B). When microbes attack plants, XEG1 is secreted early into the infected plants to degrade xyloglucan and β-glucan in plant cell walls (Ma et al., 2015; Gui et al., 2017). Wang et al. (2018) demonstrated that RXEG1 specifically recognizes XEG1, associates XEG1 via the LRR domain in the apoplast, and forms a complex with the LRR receptor-like kinases BAK1 and SOBIR1 to transduce the XEG1-induced defense signal. The mode of action for RXEG1 will help elucidate the mechanisms of disease resistance and regulation of various crops in the future. RXEG1 in *N. benthamiana* can identify XEG1 family proteins secreted by various microbes. Therefore, RXEG1 could potentially be used to protect a broad range of plants, especially other solanaceous plant species such tomatoes, whereby high disease resistance might be achieved through genetic engineering or by spraying.

Wang et al. (2018) identified a new PRR (RXEG1) and its associated PKs. Further experimentation is needed to reveal ligand binding specificity, structural requirements, and modifications of RXEG1. More specifically, studies are needed to reveal how dynamics and protein composition are controlled during immune receptor complex assembly, and which intracellular hubs are involved in transforming various microbial signal inputs into a generic plant immune response.

Currently, the recognition of certain MAMPs remains restricted to solanaceous plants (Wang et al., 2016, 2018; Franco-Orozco et al., 2017). For example, RXEG1-like genes or proteins have been found in various dicots, but not in monocots. Silencing LRR receptor candidates for identification of MAMP recognition receptors in cereal crops is a straightforward approach that would advance MAMP recognition and improve crop resistance.

## Author Contributions

WW drafted the manuscript. SW and WW revised the manuscript. SW draw Figure 1. All authors contributed to the writing of the manuscript.

### Conflict of Interest Statement

The authors declare that the research was conducted in the absence of any commercial or financial relationships that could be construed as a potential conflict of interest.
